# Brief acceptance and commitment therapy for children and adolescents with type 1 diabetes

**DOI:** 10.3389/fpsyg.2024.1382509

**Published:** 2024-06-26

**Authors:** Cristina Stefanescu, Aurel Nechita, Claudia Iuliana Iacob

**Affiliations:** ^1^Faculty of Medicine and Pharmacy, “Dunărea de Jos” University, Galați, Romania; ^2^Laboratory of Health Psychology and Clinical Neuropsychology, Department of Applied Psychology and Psychotherapy, Faculty of Psychology and Educational Sciences, University of Bucharest, Bucharest, Romania

**Keywords:** paediatric diabetes, acceptance and commitment therapy, type 1 diabetes, online act, stress, psychological flexibility

## Abstract

**Introduction:**

Children and adolescents with diabetes face challenges that can significantly impact their quality of life. Investigating psychological interventions for stress management can equip them with the skills needed to cope with type 1 diabetes. This study investigated the impact of a brief Acceptance and Commitment Therapy (ACT) intervention on stress management, diabetes acceptance, psychological flexibility, and patient-doctor relationships among this population.

**Methods:**

A total of 55 children, and adolescents from Romania participated in a four-session ACT intervention for type 1 diabetes. The evaluation instruments used were the Perceived Stress Scale for Children (PSS-C), Acceptance and Action Diabetes Questionnaire (AADQ), Children’s Psychological Flexibility Questionnaire (CPFQ), and Patient-Doctor Relationship Questionnaire (PDRQ-9).

**Results:**

The participants (mean age = 14.14, SD = 2.26; 67% girls) reported lower stress levels, increased acceptance of diabetes, and greater psychological flexibility after the intervention. Additionally, the patient-doctor relationship was enhanced, potentially improving patient adherence to treatment. Positive qualitative feedback mirrored previous ACT research in the paediatric population, highlighting the beneficial role of experiential activities and metaphors when working with this target group.

**Discussion:**

This study adds to the growing body of evidence supporting the effectiveness of ACT in enhancing healthy coping strategies among children and adolescents with chronic illnesses.

## 1 Introduction

Diabetes, a chronic condition that can significantly impair bodily function, also leads to psychological distress (World Health Organization, 2023). While it affects both children and adults, childhood diabetes is often unrecognised by parents (Howe et al., 2015; Chikani et al., 2018). This can lead to delays in diagnosis and treatment, exacerbating the child’s condition (Royal College of Paediatrics and Child Health, 2020).

The main types are type 1, which requires insulin injections, and type 2, which can be managed with medication and lifestyle changes. The importance of glycaemic management in type 1 diabetes (T1D) complications has been well established, especially monitoring the percentage of time a person spends with the glucose level in a targeted range (Yapanis et al., 2022; Suh et al., 2023). These complications are linked to the intensity and duration of hyperglycaemia, as measured by glycaemic control indicators. Managing T1D involves a challenging lifestyle, particularly due to insulin injections, which are especially difficult for children. Early identification and effective management are crucial given the rising prevalence and incidence of type 1 and type 2 diabetes among children and adolescents in the US and Europe (Candler et al., 2018; Patterson et al., 2019; Lawrence et al., 2021; Cohen et al., 2022). In 2021, T1D affected approximately 844 million people globally, including 15 million (18%) under 20 years of age, highlighting its significant impact on children (Gregory et al., 2022).

Children and adolescents with diabetes often face overwhelming feelings due to daily management demands, societal perceptions, and the challenges of leading lives like their peers (Streisand and Monaghan, 2014). They require frequent blood glucose monitoring, insulin administration, and diet and exercise control. Despite parental involvement, patient responses can be suboptimal (Sullivan-Bolyai, 2004). Adherence to treatment is closely linked to patient satisfaction and effective communication with doctors (Zolnierek and DiMatteo, 2009). Establishing a functional patient-doctor relationship (PDR) is particularly challenging for adolescents (Lowes et al., 2004, 2015). Thus, investigating psychosocial interventions to improve diabetes coping mechanisms and PDR is essential. The psychological flexibility framework (Hayes et al., 2012) has recently gained attention in this direction.

### 1.1 Psychological flexibility, acceptance and commitment therapy (ACT) and paediatric T1D management

Psychological flexibility, defined as the ability to be aware of the present moment and adjust behaviour according to personal values (Hayes et al., 2006), is important for managing chronic conditions like T1D. The Psychological Flexibility Model (Hayes et al., 2012) includes six processes: present-moment awareness, acceptance (willingness to experience thoughts or emotions without controlling them), cognitive defusion (observing thoughts without becoming entangled), self-context (seeing oneself beyond thoughts and feelings), values, and committed action. This model is the foundation of ACT, a third-wave cognitive-behavioural and process-based therapeutic approach (Hayes et al., 2012; Hayes and Hofmann, 2021) that has shown promise in improving psychosocial and health outcomes in adolescents with T1D by reducing psychological symptoms and physical health complaints (A-Tjak et al., 2015).

ACT interventions have been effective in chronic conditions such as type 1 and type 2 diabetes, fibromyalgia, and chronic pain by improving acceptance, adherence, mood, and reducing pain (Wicksell et al., 2013; Alho et al., 2022, 2024). A meta-analysis of 212 studies (Compas et al., 2017) has examined the impact of coping methods on psychological outcomes in response to stress. The results showed that acceptance-based coping processes significantly reduced psychological symptoms in children and adolescents both cross-sectionally and longitudinally. In contrast, avoidant coping led to increased psychological distress. These findings support the importance of ACT in enhancing stress management and emotion-regulation skills. Another meta-analysis (Fang and Ding, 2020) reviewed seven randomised controlled trials of ACT in children aged ≤12 years. The results showed that ACT significantly reduced anxiety and sadness, comparable to cognitive-behavioural therapy (CBT). Three studies highlighted ACT’s effectiveness in improving symptoms, quality of life, and psychological flexibility in children, with similar outcomes in follow-up assessments (Swain et al., 2015).

Children generally responded positively to ACT, particularly appreciating the interactive aspects of the six processes of psychological flexibility, which makes ACT a suitable intervention for this target group. Improvements in parental well-being and mindfulness have also been shown to indirectly enhance children’s QoL (Sairanen et al., 2022).

The literature on ACT and T1D is an emerging field (Iina et al., 2021; Alho et al., 2022, 2024), and available evidence suggests the utility of ACT in young individuals with T1D. Overall, previous studies investigated psychological flexibility, quality of life, acceptance, anxiety, and depression, but they did not target PDR, which is linked to treatment adherence (Zolnierek and DiMatteo, 2009). There is a gap in the research specifically focusing on the impact of ACT on stress management and PDR in this target group. To address this gap and support some of the previous findings, we examined the impact of a brief individual ACT protocol on stress, diabetes acceptance, psychological flexibility, and PDR in a group of children with T1D.

## 2 Methods

### 2.1 Participants and design

Fifty-five children and adolescents from Romania participated in this single-arm study. The study included children and adolescents diagnosed with T1D for at least 1 year, with a stable treatment plan, and without additional comorbid medical diagnoses that could potentially impact cognitive functioning (such as intellectual disability). Individuals with significant cognitive impairment, severe and persistent psychiatric conditions such as schizophrenia or bipolar affective disorder, life-threatening physical conditions, or those currently receiving psychological support were excluded from the study. These criteria were established to ensure the homogeneity of participants.

The participants, aged between 10 and 18 years, were recruited from two sources: patients hospitalised at St. John Emergency Hospital for Children in Galați, and members of online support groups for diabetes. Hospital admissions were aimed at adjusting and maintaining proper glucose levels in those already diagnosed.

### 2.2 Instruments

Stress was measured using the Perceived Stress Scale for Children (PSS-C; White, 2014). This self-report measure provides a global stress score based on broad questions rather than individual events. The participants were asked to rate their stress levels in the previous month. The scale has 14 items scored on a four-point Likert scale ranging from 1 to 4. The total score was obtained by summing the answers on all items (seven items had reverse scoring). Higher total scores indicate higher stress levels (White, 2014). The Cronbach’s alpha for consistency in this study was 0.77.

Psychological flexibility was assessed using the Children’s Psychological Flexibility Questionnaire (CPFQ; Lenoir et al., 2022), a 24-item self-report measure completed by children. The items were organised according to hexaflex. The following items were reversed: 11 and 18 (present moment); 4 and 21 (self-as-context); 8 and 19 (acceptance); 7 and 23 (values); 13 and 20 (defusion); and 6 and 17 (committed action). All responses were combined to obtain the total psychological flexibility scores. Lower scores imply greater psychological inflexibility, whereas higher scores indicate better psychological flexibility. The Cronbach’s alpha for this study was 0.78.

Diabetes acceptance was evaluated using the Acceptance and Action Diabetes Questionnaire (AADQ; Gregg et al., 2007). The AADQ has six items and asks respondents to use a 5-point Likert scale (1 = “Never” to 5 = “Always”) to indicate how much they engage in diabetes non-acceptance behaviours (e.g., “I try to avoid reminders of my diabetes,” “When I have an upsetting feeling or thought about my diabetes, I try to get rid of that feeling or thought,” “I avoid thinking about what diabetes can do to me”). Lower ratings indicate a higher level of acceptance. The Cronbach’s alpha for this study was 0.95.

The patient-doctor relationship was measured using the Patient-Doctor Relationship Questionnaire (PDRQ-9; van der Feltz-Cornelis et al., 2004), a 9-item scale that contains questions concerning helpfulness (e.g., “My doctor helps me”), trustworthiness (e.g., “I trust my doctor”), comprehension (e.g., “My doctor understands me”), devotion, and accessibility of the doctor. Responses were scored on a 5-point Likert scale, with 0 representing “not at all appropriate” and 4 representing “totally appropriate.” The total score is obtained by summing the answers. Higher scores reflected a better patient-doctor relationship. The Cronbach’s alpha for this study was 0.98.

All instruments used in this study were subjected to simple translation from English to Romanian, followed by back-translation by two PhD psychology researchers. In addition, the participants provided basic sociodemographic information (age, sex, and living area). At the end of the last session, each participant was asked to provide verbal feedback on their experiences with the ACT sessions. The researcher recorded their responses during the session.

### 2.3 Intervention

The study incorporated a 4 - week brief ACT intervention designed for children and adolescents. The intervention was tailored according to Black (2022) recommendations for ACT in the paediatric population. Each session lasted approximately 50 min and was delivered individually by a clinical psychologist (also a PhD researcher in medicine). During the first session, the ACT model was introduced and participants were asked about their coping mechanisms when facing stress-related thoughts about diabetes and daily tasks. The importance of adaptability in uncertain circumstances, encompassing aspects of life with diabetes, interactions with healthcare professionals, and adherence to treatment, is discussed. The session ended with a mindfulness activity aimed at fostering the present-moment skills and emotion regulation.

The second session focused on exploring their fear of making mistakes. A Romanian therapeutic story illustrating Dora’s fear of making mistakes (Filipoi, 1998) was used, along with guided imagery techniques, to enhance cognitive defusion skills. Guided imagery and the “clouds in the sky” metaphor (Black, 2022) were also introduced to practice cognitive defusion.

During the third session, a grounding exercise was conducted to enhance contact with the present moment, and the participants had the opportunity to explore their needs. Some participants wanted to delve deeper into cognitive defusion processes through another guided imagery activity, whereas others wanted to talk about creating a space between themselves and their T1D. They elaborated on their problems, such as communicating with doctors and integrating diet and exercise into their schedule. Personal values and how to use what matters the most to achieve self-declared goals were addressed.

In the final session, the psychologist emphasised the significance of mindful eating and the need to adopt healthy eating habits to cultivate the ability to fully appreciate the present moment in all aspects of life, particularly during mealtimes. Participants engaged in discussions about physicians’ recommendations regarding their eating habits and reflected on their perception of these recommendations. At the end of the session, the participants were invited to ask questions and provide feedback regarding the intervention.

### 2.4 Procedure

Participants were recruited through online channels such as Romanian diabetes groups, social media platforms, and recommendations from St. John Hospital for Children, Galați, Romania between December 2022 and December 2023. The study was conducted in compliance with the Declaration of Helsinki and approved by the Ethics Committee of St. John Hospital for Children Galați, Romania (notice no. 35792/29.12.2022).

Prior to participation, written informed consent was obtained from the parents or legal guardians of all the participants. Additionally, written assent was obtained from the children and adolescents themselves to ensure they understood the nature of the study and their involvement. The consent and assent forms were designed to be age-appropriate and clearly explain the study’s purpose, procedures, potential risks, and benefits. Participants completed pre- and post-intervention behavioural measures using Google Forms. Face-to-face ACT sessions (33% of participants) were conducted at St. John Emergency Hospital for Children Galați, Romania and online sessions via the Zoom platform (67% of participants) were also used. All collected data were anonymised and securely stored to ensure confidentiality, in line with the General Data Protection Regulation (GDPR; Directive 95/46/CE). Participation was voluntary, unconditional, and without any consequences of dropping out. Participants were reminded of their right to withdraw from the study at any point, and data collected up to the point of withdrawal remained a part of the study.

The calculations performed using G*Power 3.1.9.7 (Faul et al., 2009) revealed that a minimum of 54 participants were required to detect mean differences between two matched pairs. The power analysis utilised a paired t-test (two-tailed), assuming a medium effect size (*d* = 0.5), an alpha error probability of 0.05, and a power (1–β) of 0.95.

### 2.5 Statistical analyses

Data were analysed using IBM SPSS Statistics version 25 (IBM Corp, 2017). First, the main descriptive statistics for the investigated variables (mean, standard deviation, skewness and kurtosis as normality indicators, and Pearson’s correlation coefficients) were reported. Paired sample t-tests and the Wilcoxon signed-rank test were used to assess pre- and post-intervention differences. A paired samples t-test was conducted for variables that met the assumption of normality, and the Wilcoxon signed-rank test was used for the other variables.

The normality of the distribution was verified using the [−1.96; +1.96] interval as the cut-off for normality indicators (George and Mallery, 2010). The minimum significance threshold was set at 0.05. Effect sizes for the paired sample t-test were calculated using Cohen’s d, which is a measure of the magnitude of the effect of the intervention. Cohen’s d values are interpreted: 0.2 (small effect), 0.5 (medium effect), and 0.8 (large effect; Cohen, 1988).Finally, we summarised the narrative feedback received from the participants regarding their experiences with the ACT sessions.

## 3 Results

Initially, 57 participants were enrolled in this study. Hospitalised patients who met the inclusion criteria were invited to participate in the study face-to-face. However, two participants dropped out when their hospitalisation ended, and they chose not to continue the intervention online. The final sample included 55 children and adolescents, with a mean age of 14.14 (sd = 2.26). The CONSORT flow chart is available in the Supplementary material. Most of the participants (67%) were girls. Almost all participants lived in urban areas.

Table 1 presents the Pearson correlations and the main descriptive indicators of the variables measured before and after the intervention. Prior to the intervention, a significant correlation was observed between stress, diabetes acceptance, and patient-doctor relationship. Participants who reported higher levels of stress tended to have lower acceptance levels and weaker patient-doctor relationships. Following the intervention, only the correlation between diabetes acceptance and patient-doctor relationship remained statistically significant. Diabetes acceptance and patient-doctor relationship post-intervention scores showed deviations from the normal distribution.

**Table 1:** Descriptive statistics of measured variables.

	Pre intervention				Post intervention			
	Stress	Flexibility	Acceptance	PDR	Stress	Flexibility	Acceptance	PDR
Stress	–				–			
Flexibility	0.03	–			0.08	–		
Acceptance	0.75**	0.06	–		0.18	−0.10	–	
PDR	−0.73**	−0.07	−0.75	–	−0.18	−0.18	−0.27*	–
Mean	36.27	46.05	24.35	18.96	25.91	56.07	8.53	33.02
SD	(7.80)	(14.75)	(7.93)	(10.34)	(6.01)	(13.07)	(4.39)	(5.74)
Skewness	−0.15	−0.19	−1.09	0.72	0.60	0.22	1.81	−2.38
Kurtosis	−0.78	0.64	−0.36	−0.72	−0.77	0.16	2.28	6.44

PDR, patient-doctor relationship; SD, standard deviation for the means of the variables; *p < 0.05, **p < 0.01.

Pre- and post-intervention measurements showed a statistically significant difference between stress (*t*(54) = 8.75, *p* < 0.001, *d* = 1.18) and psychological flexibility (*t*(54) = −4.00, *p* < 0.001, *d* = −0.54) before and after the ACT sessions. In other words, stress levels decreased and psychological flexibility increased, as expected (Table 2).

**Table 2:** Pre- and post-intervention differences regarding stress and psychological flexibility.

Variable	95% Confidence interval of the difference		t(54)	*p*	Effect size (Cohen’s d)
	Lower	Upper			
Stress	0.83	1.52	8.75	< 0.001	1.18
Psychological flexibility	−0.82	−0.25	−4.00	< 0.001	−0.54

The data also showed that diabetes acceptance increased post-intervention (*Z* = 1417.5, *p* < 0.001, *r* = 0.98), and PDR significantly improved (*Z* = 89.5, *p* < 0.001, *r* = −0.86; Table 3), with large effect sizes. Thus, the intervention had a substantial impact on the variables.

**Table 3:** Pre- and post-intervention differences regarding diabetes acceptance and patient-doctor relationship.

Variable	Wilcoxon test W	*p*	Pre- and post-intervention mean differences	Effect size (r)
Diabetes acceptance	1417.5	<0.001	15.8	0.98
PDR	89.5	<0.001	−15.5	−0.86

PDR, patient-doctor relationship.

At the end of the sessions, participants were asked to provide feedback on the intervention. Overall, narrative feedback suggests that the intervention helped participants improve their emotional awareness, cope with challenging emotions, and foster a more accepting view of their interactions with healthcare professionals. For example, some children were very interested in the sessions, reporting that the stories brought them a lot of joy and that they liked to learn how to name and stay with their emotions. A 10-year-old girl reported, “*I was reluctant to do the sessions with you at first, because I did not know what a psychologist does. Now, I can identify, name, and describe my emotions and know what to do when I feel angry with myself and the world around me, especially with doctors. Doctors seemed friendlier after you told me about how the patients’ emotions had an impact on them as well*.” A 12-year-old boy also commented on the patient-doctor relationship after the sessions: “*Doctors are human beings and they have bad days. It is okay if they do not recognise me on the hallway or if they do not say hello or remember my name. They have many patients and I learned during our talks to accept this.*”

A 14 years old girl said that her favourite story was one in which she had to reflect on her own mistakes and make peace with them:” *I felt that it was ok to make mistakes and to acknowledge them, learn from them, make space and tolerate difficult emotions related to things that I regret*.” Another participant, a 15 year-old girl, gave the following feedback: “*There are some patients who complain all the time about hospitals, doctors, medication and so on and so forth; after talking to you I realized that nothing really is perfect in this world and that I should always fallow what I love and value and use them as motivation and focus on that instead on what is wrong with this world. When I started focusing on being healthy instead of being right the whole situation changed*.”

## 4 Discussion

This study aimed to investigate the impact of a four-week individual ACT intervention in children and adolescents with T1D in Romania. After the intervention, the participants reported significantly lower levels of stress, greater acceptance of diabetes, and greater psychological flexibility compared to the pre-intervention assessment. In addition, the PDR improved post-intervention.

Even though studies conducted on paediatric diabetes and ACT are scarce (Iina et al., 2021; Alho et al., 2022, 2024), our findings are congruent with the existing literature and support ACT as a promising intervention for supporting T1D management in children and adolescents. Moreover, published data on adults with diabetes also highlight ACT’s role in improving the quality of life and mental health-related outcomes in patients with type 1 and type 2 diabetes (Sakamoto et al., 2022; Somaini et al., 2023). The transdiagnostic nature of ACT and the individual format in which the intervention was delivered favoured the personalisation of therapeutic dialogue and touching on topics relevant to the participants’ lives. As such, psychoeducational content could be adapted to the participants’ situation, even though illness-related elements were common. It was easier for children and adolescents to talk about their concerns and doctor-patient relationships when protected by confidentiality.

The intervention targeted the psychophysiological, cognitive, emotional, and behavioural components of human functioning. Using mindfulness activities, participants learned to manage the stress related to the chronic nature of their condition. This, in turn, can help them to better control their glycaemic levels (Argyropoulos et al., 2021; Inverso et al., 2022). For children with diabetes, who may experience stress related to concerns about their health, cognitive defusion skills create a distance from disturbing thoughts, making it easier to cope with them (Hayes et al., 2012). At an emotional level, living with a chronic illness brings challenges such as anxiety and depression (Akbarizadeh et al., 2022). Therefore, working on accepting emotions and conditions, coupled with value-based behaviours, can reduce distress and make room for a better life.

The novelty of this study is ACT’s potential to optimise PDR, which, in the long run, could have a beneficial impact on patient adherence to treatment (Zolnierek and DiMatteo, 2009; Patel et al., 2018). Future research can replicate this result, and further data are needed to determine whether psychological flexibility mediates or moderates the relationship between stress and the PDR.

Qualitative feedback from the participants was consistent with previous studies on ACT and the paediatric population. For example, Clery et al. (2021) conducted a qualitative study on ACT acceptability in teenagers with chronic fatigue syndrome, and found that all participants and their parents considered ACT acceptable. Furthermore, some participants in Clery et al. (2021) study preferred ACT to CBT, because the defusion component replaced cognitive restructuring. They used words such as „more gentle and kinder” to describe ACT in relation to CBT (Clery et al., 2021). Although it was not possible to compare ACT and CBT in our study, feedback regarding ACT is congruent with the literature. Some participants expressed a heightened interest in the sessions, noting that the therapeutic stories brought them joy. Reported outcomes include improved emotional awareness with children learning to identify, name, and describe their emotions. One participant highlighted a transformation in their perception of psychologists, initially being hesitant but later appreciating their ability to navigate and manage emotions, especially in challenging situations such as interactions with doctors.

Insights into the patient-doctor relationship have also emerged from qualitative feedback, emphasising doctors’ humanity and acceptance of their limitations, particularly in terms of recognising every patient in a busy hospital environment. Participants gained a more empathetic perspective and understood that doctors, like anyone else, could have bad days. These verbal reactions complement the quantitative answers from the PDRQ (van der Feltz-Cornelis et al., 2004), where significant improvements were noted post-intervention, and offer possible mechanisms of change in the patient-doctor relationship for this particular group of participants. The intervention had an impact on how the participants approached mistakes, with one girl expressing acceptance, learning, and peace with her mistakes, as she reported after the sessions. Another participant emphasised the importance of focusing on personal values and motivations rather than dwelling on the imperfections of the world, leading to a positive shift in their overall perspective, particularly regarding health and well-being. The participants’ narrative feedback was consistent with the quantitative assessment, which showed lower stress levels, higher psychological flexibility, acceptance of diabetes, and improved PDR.

The development of evidence-based multidisciplinary management guidelines for paediatric patients with diabetes that incorporate psychological interventions informed by the ACT model is proposed in light of our findings and the literature. There is a significant gap in Romania where no such guidelines are currently available. Establishing these guidelines would greatly benefit health care professionals in their efforts to deliver optimal care to these patients.

### 4.1 Strengths and limitations

Overall, this study contributes to recent evidence showing the usefulness of ACT in improving quality of life and mental health-related variables in children and adolescents with chronic illnesses (Fang and Ding, 2020; Iina et al., 2021). Additionally, it recommends using this intervention to optimise PDR. One of the strengths of this study is its focus on a relatively underexplored population: children and adolescents with T1D in Romania. By targeting this demographic group, the study addresses a gap in the existing literature on paediatric diabetes management. Another strength is the incorporation of both quantitative and qualitative measures to investigate the intervention’s outcomes. Moreover, the impact of ACT on PDR was not explored to date in this target group and the current study provided initial evidence of ACT’s contribution in this direction.

At the same time, the results should be considered in light of the limitations of this study. First, no control group was used, which diminished the methodological rigour of the study. Psychotherapy and counselling in Romania are still not a popular practice, and stigma and negative stereotypes are associated with them (Manescu et al., 2023), making it difficult to motivate people to participate in such studies. Second, the sampling procedure and small sample size, though sufficient for power analysis, limit the generalisability of the findings. However, this study is a step towards building evidence in the emerging research area of ACT for children and adolescents with T1D. Further research with larger samples is necessary to confirm these results and to enhance their applicability. Third, the study did not account for the homogeneity of the participants regarding glycaemic control. Future research should evaluate and ensure comparable glycaemic control levels among participants to better isolate the effects of the intervention. Fourth, the measurements relied only on children’s perceptions and no parent reports were included. Finally, no follow-up assessment of the long-term effects of the intervention was performed.

## Data availability statement

The raw data supporting the conclusions of this article will be made available by the authors, without undue reservation.

## Ethics statement

The studies involving humans were approved by Ethics Committee of St. John Hospital for Children, Galați, Romania (notice no. 35792/29.12.2022). The studies were conducted in accordance with the local legislation and institutional requirements. Written informed consent for participation in this study was provided by the participants’ legal guardians/next of kin.

## Author contributions

CS: Writing – review & editing, Writing – original draft, Methodology, Investigation, Conceptualization. AN: Writing – original draft, Validation, Project administration, Investigation, Conceptualization. CI: Writing – review & editing, Methodology, Formal analysis, Data curation.
