# Supplementary material for: Brief acceptance and commitment therapy for children and adolescents with type 1 diabetes
Source: Front Psychol. 2024 Jun 26;15:1382509. doi: 10.3389/fpsyg.2024.1382509 (PMC11234476; doi:10.3389/fpsyg.2024.1382509)
Supplement: Supplementary file 1 [file Table_1.DOCX]

Supplementary Material

Assessed for eligibility (n = 57)

Enrollment

Excluded (n = 2)

- Withdrew from participation after initial consent, before the intervention started (n = 2)

Recruited (n = 55)

**Supplementary Figure 1.** The CONSORT flow diagram
